# Genetic Effects on Toxic and Essential Elements in Humans: Arsenic, Cadmium, Copper, Lead, Mercury, Selenium, and Zinc in Erythrocytes

**DOI:** 10.1289/ehp.0901541

**Published:** 2010-01-05

**Authors:** John B. Whitfield, Veronica Dy, Robert McQuilty, Gu Zhu, Andrew C. Heath, Grant W. Montgomery, Nicholas G. Martin

**Affiliations:** 1 Genetic Epidemiology Unit, Queensland Institute of Medical Research, Brisbane, Australia; 2 Biochemistry Department, Royal Prince Alfred Hospital, Sydney, Australia; 3 Midwest Alcoholism Research Center, Washington University, St. Louis, Missouri, USA

**Keywords:** arsenic, cadmium, copper, erythrocytes, genetics, lead, mercury, selenium, twins, zinc

## Abstract

**Background and objectives:**

An excess of toxic trace elements or a deficiency of essential ones has been implicated in many common diseases or public health problems, but little is known about causes of variation between people living within similar environments. We estimated effects of personal and socioeconomic characteristics on concentrations of arsenic (As), cadmium (Cd), copper (Cu), mercury (Hg), lead (Pb), selenium (Se), and zinc (Zn) in erythrocytes and tested for genetic effects using data from twin pairs.

**Methods:**

We used blood samples from 2,926 adult twins living in Australia (1,925 women and 1,001 men; 30–92 years of age) and determined element concentrations in erythrocytes by inductively coupled plasma-mass spectrometry. We assessed associations between element concentrations and personal and socioeconomic characteristics, as well as the sources of genetic and environmental variation and covariation in element concentrations. We evaluated the chromosomal locations of genes affecting these characteristics by linkage analysis in 501 dizygotic twin pairs.

**Results:**

Concentrations of Cu, Se, and Zn, and of As and Hg showed substantial correlations, concentrations of As and Hg due mainly to common genetic effects. Genetic linkage analysis showed significant linkage for Pb [chromosome 3, near *SLC4A7* (solute carrier family 4, sodium bicarbonate cotransporter, member 7)] and suggestive linkage for Cd (chromosomes 2, 18, 20, and X), Hg (chromosome 5), Se (chromosomes 4 and 8), and Zn {chromosome 2, near *SLC11A1* [solute carrier family 11 (proton-coupled divalent metal ion transporters)]}.

**Conclusions:**

Although environmental exposure is a precondition for accumulation of toxic elements, individual characteristics and genetic factors are also important. Identification of the contributory genetic polymorphisms will improve our understanding of trace and toxic element uptake and distribution mechanisms.

Many investigators have considered the toxic effects of exposure to metals such as arsenic (As), cadmium (Cd), lead (Pb), and mercury (Hg), and there has been substantial expenditure on measures to reduce human exposure. Although toxic effects are clearly established for occupationally exposed people and for people in locations where particularly high levels are present, demonstration of harmful effects population-wide has been more difficult. One possible reason is that at comparatively low levels of exposure, only some people are at risk, and this makes an association more difficult to find (Bellinger 2008). Study of the causes of individual variation could improve the power of epidemiological studies to detect harm to susceptible people.

Chronic exposure to As has been associated with diabetes (Coronado-Gonzalez et al. 2007; Navas-Acien et al. 2006), cardiovascular disease (Wang et al. 2007), intellectual impairment in children (von Ehrenstein et al. 2007; Wasserman et al. 2007), and lung and bladder cancers (Navarro Silvera and Rohan 2007). Cd primarily affects the kidneys, with tubular proteinuria providing a useful marker of the renal damage. Studies in Cd-polluted areas have reported an excess mortality risk among people showing tubular or glomerular proteinuria, but the causes of this excess mortality are unclear (Satarug et al. 2003). Other claimed adverse effects of Cd include low bone-mineral density and osteoporosis (Alfven et al. 2000, 2004) and lung cancer (Navarro Silvera and Rohan 2007). Hg has been implicated in severe but localized poisonings (Ekino et al. 2007). Widely publicized concerns about effects of Hg from vaccine preservatives have been refuted (Thompson et al. 2007), but effects on intellectual functioning or behavior have been reported or suggested (Axelrad et al. 2007; Davidson et al. 2004). Cardiovascular (Virtanen et al. 2007) and renal (Hodgson et al. 2007) disease have been associated with low-level Hg exposure. For Pb, the main concerns have been about impaired intellectual development in infancy and childhood (Canfield et al. 2003; Needleman 2004; Pocock et al. 1994; Schwartz 1994), but adult cognitive function may also be affected (Schwartz and Hu 2007; Shih et al. 2007). There is also evidence that higher Pb values are associated with hypertension (Navas-Acien et al. 2007), peripheral vascular disease (Navas-Acien et al. 2004), increased adult mortality (Lustberg and Silbergeld 2002; Schober et al. 2006), and cognitive decline in older people (Weisskopf et al. 2004). Studies on overall and cause-specific mortality suggest a 40–60% increase in adjusted mortality with increasing Pb concentration in the U.S. population (Jemal et al. 2002; Lustberg and Silbergeld 2002; Menke et al. 2006; Schober et al. 2006).

Exposure is a precondition for accumulation of these elements. There is undoubtedly individual variation in exposure, but this may be only one of several factors influencing toxic element accumulation and body burden. Identification of “host factors” has the potential to clarify the relationships between exposure and harm. These may act through variation in absorption or distribution within the body, brought about by differences in behavior (such as smoking and alcohol consumption), or by genetic differences in metabolic or biotransformation processes. Furthermore, even at a uniform level of cell or tissue exposure there may be differences in response because of variation in repair mechanisms, antioxidant status, availability of protective elements, or other so far unrecognized factors.

Several attempts have been made to test for effects of genetic polymorphisms on toxic element accumulation, particularly using blood or bone Pb values. So far there are mixed results, with allelic associations both reported and denied for the *ALAD* (δ-aminolevulinic acid dehydratase) and *VDR* [vitamin D (1,25-dihydroxyvitamin D3) receptor] genes. More generally, only a few studies (Bjorkman et al. 2000; Hopper et al. 1982; Hopper and Mathews 1983; Whitfield et al. 2007) have considered the question of familial similarity, and most have only examined Pb. We recently reported a twin study that showed significant genetic effects on blood Pb concentration (Whitfield et al. 2007). However, in the literature, we found no similar data for the other toxic elements, apart from one study that examined both Pb and Cd (Bjorkman et al. 2000).

Current analytical methods allow the simultaneous measurement of multiple elements in biological samples, including both the potentially toxic ones already mentioned and essential elements including copper (Cu), selenium (Se), and zinc (Zn). These are not only necessary for function of some enzymes, but they potentially interact with the absorption, distribution, or effects of the toxic elements (Peraza et al. 1998).

We have now examined data on As, Cd, Hg, and also the essential elements Cu, Se, and Zn in blood samples from twin subjects previously studied for Pb. Our primary aim was to evaluate the contributions of genetic and environmental factors to interindividual differences. We have also assessed the contributions of physiological or demographic characteristics to variation, estimated correlations between concentrations of these elements, and performed linkage analysis using data from dizygotic (DZ) twin pairs. We aimed to determine which characteristics affect the uptake or distribution of these potentially toxic elements; whether elements other than Pb show evidence for genetic effects; and to what degree the correlations between concentrations of these elements are due to environmental effects (such as simultaneous exposure to more than one potentially toxic element in adverse environments) or to genetic effects (such as variation in uptake or distribution kinetics affecting multiple elements through common biological mechanisms); and to identify gene loci that may affect other elements, as we previously have for Pb.

## Materials and Methods

### Participants

Participants were twins enrolled in the Australian Twin Registry and born between 1903 and 1964. The subjects and procedures were the same as those described previously for blood Pb (Whitfield et al. 2007). Briefly, participants completed a postal questionnaire in 1989, completed a telephone interview in 1993–1994, and provided a blood sample in 1993–1996 (Whitfield et al. 1998). We determined zygosity from responses to questions about physical similarity and the inability of others to tell them apart, supplemented by blood group information and, for those DZ pairs participating in linkage projects (Beekman et al. 2003), genome-wide microsatellite genotyping. Details are given in the Supplemental Material (doi:10.1289/ehp.0901541). Participants gave written informed consent, and the studies were approved by the appropriate ethics committees.

In 1993–1996, blood was collected from 1,134 men and 2,241 women. At the same visit, each participant’s height and weight were measured and body mass index (BMI) was calculated (weight in kilograms divided by height in meters squared). Information on alcohol intake, smoking, years of education, and social class was obtained by self-report questionnaires, as described in the Supplemental Material (doi:10.1289/ehp.0901541). Participants’ addresses were categorized into urban, suburban, or rural zones using their postcodes at the time of blood collection and a database on the geographic location of Australian postcodes. Information on subjects is summarized in Supplemental Material, Table 1.

### Laboratory procedures

Because plasma and buffy from the original blood samples were used for other purposes, we used erythrocytes for elemental analysis. Before analysis, the erythrocytes were thawed at room temperature and diluted 1:20 in ammonia/EDTA solution containing rhodium as an internal standard. As, Cd, Cu, Hg, Pb, Se, and Zn concentrations were measured by inductively coupled plasma mass spectrometry on a Perkin-Elmer Elan 5000 mass spectrometer (PerkinElmer Inc, Wellesley, MA, USA) or a Varian UltraMass (Varian Inc., Palo Alto, CA, USA). Hemoglobin concentration was then measured on the diluted samples using the cyanmethemoglobin method. Quality control methods and results are discussed in the Supplemental Material (doi:10.1289/ehp.0901541).

### Data analysis

We measured erythrocyte element concentrations for 2,926 individuals. We accounted for error variation inherent in measurement of element concentrations in the samples, with both quality control and twin samples contributing to the maximum-likelihood estimation of and adjustment for day effects [see Supplemental Material (doi:10.1289/ehp.0901541) for additional details].

We performed model fitting to test for effects of genetic and environmental sources of variation and multivariate analysis of covariate effects using Mx (Neale 1999). Data from 2,832 people were analyzed using Mx: 428 monozygotic (MZ) female, 165 MZ male, 218 DZ female, 90 DZ male, and 222 DZ opposite-sex pairs; and 586 individuals (356 female and 230 male) whose co-twin did not participate. The latter contribute to estimates of means and variances but not directly to co-twin correlations.

### Effects of covariates

We adjusted for sample hemoglobin concentration, effects of day-to-day analytical variation, sex, and age. At each step, the improvement in goodness-of-fit was assessed by the likelihood ratio chi-square test as each variable was added. We then tested for the effects of personal, geographic, and social characteristics expected to affect the results: sex, age, alcohol intake, smoking status, residential location, social class, educational level, and BMI. Univariate analysis using SPSS (SPSS Inc., Chicago, IL, USA) was supplemented with Mx analysis to assess the independent effects of these covariates and to take account of twin relatedness. The baseline model contained all the covariates; a series of submodels were then fitted in which one of the covariates was removed and the change in goodness-of-fit calculated to determine the significance of their independent contribution. See Supplemental Material (doi:10.1289/ehp.0901541) for additional details.

### Genetic and environmental sources of variation

We saved residuals from the model containing all the covariates and used them to fit models of genetic and environmental sources of variation and covariation to the within- and between-pair covariances by zygosity group, as previously described (Whitfield et al. 2007), and also for the linkage analysis. For consistency (and because we particularly wished to distinguish between shared environmental and genetic effects), we initially tested models with additive genetic (A), nonshared environmental (E), and shared environmental (C) sources of variation; these ACE models were then compared with models containing only A and E, to determine whether shared environmental sources of variation could be excluded.

### Linkage analysis

We performed linkage scans for erythrocyte element concentrations on 501 DZ twin pairs with phenotypic data and linkage marker data. DNA was extracted from blood or buccal swabs according to standard procedures (Miller et al. 1988). Genotyping data were collated from genome-scans previously performed for other projects. Errors were resolved and Mendelian segregation inconsistencies were identified and removed as described in Supplemental Material (doi:10.1289/ehp.0901541).

Of the 501 available twin pairs, 487 (97%) were genotyped at 300–1,717 markers (mean, 743) and 14 (3%) at < 300 markers. Sibling identity by descent was estimated as described in Supplemental Material (doi:10.1289/ehp.0901541).

### Empirical genome-wide thresholds

For the Pb-linkage peak, we estimated the probability of the observed logarithm of odds (LOD) score occurring by chance using 1,000 gene-dropping simulations as described by Abecasis et al. (2004). See Supplemental Material (doi:10.1289/ehp.0901541) for additional details.

In summary, we measured element concentrations (As, Cd, Cu, Hg, Pb, Se, Zn) in erythrocytes from the blood samples. We estimated heritability of erythrocyte concentration for each element from the MZ and DZ twin-pair data after testing for effects of covariates [listed in Supplemental Material, Table 2 (doi:10.1289/ehp.0901541)], adjusting for covariate effects. Chromosomal locations of gene variants affecting element concentrations were evaluated using genome-wide linkage analysis.

## Results

### Covariate effects

We observed significant associations of sex with Cd, Cu, and Se (all with higher concentrations in women) and on Pb (with higher concentrations in men). Pb and Se increased significantly with age. Reported alcohol consumption was significantly associated with increases in As, Hg, Pb, and Se [see Supplemental Material, Figure 1 (doi:10.1289/ehp.0901541)]. Increasing self-reported alcohol intake was also associated with increasing Cd, but this was due to the strong association between smoking and higher alcohol intake. Smoking had very substantial positive associations with Cd, as expected, and with Hg, Pb, and Se. Of the demographic factors, body mass index and social class had no significant associations, whereas educational level was inversely associated with As, Cd, and Pb. Place of residence was associated with Hg and Zn; Hg was higher in urban residents and Zn higher in rural residents. See Supplemental Material, Table 2 for a summary of significant effects of the covariates on element concentrations.

### Element correlations

We observed significant correlations between concentrations of several of the elements in both men and women, after adjusting for day-of-analysis effects, quality control data, and sample hemoglobin concentration (Table 1). There were substantial associations (*r* ≈ 0.4–0.5) between Cu, Zn, and Se and between As and Hg. Correlations between Pb and Cu, Zn, and Cd and for Se with As and Hg were more moderate, but still significant (*r* ≈ 0.2). Results for men and women were similar.

### Sources of variation and covariation

The pairwise correlations for the measured elements for MZ and DZ twin pairs are shown in Table 2. In each case the MZ correlation was greater than the DZ correlation, and for all except Cd the 95% confidence intervals (CIs) did not overlap. The proportion of variation in each element accounted for by additive genetic factors (A), environments shared by co-twins regardless of zygosity (C), and nonshared environments including measurement error and within-person biological variation (E), are shown in Table 2. These estimates of A, C, and E were derived under the ACE model allowing for all three sources of variation, but models containing A and E only did not show a significantly worse fit (all *p* > 0.05).

Because of our interest in genetic or environmental sources of variation affecting more than one element, we re-estimated the genetic and environmental sources of variation in a multivariate ACE model [see Supplemental Material, Table 3 (doi:10.1289/ehp.0901541)]. Minor differences from the univariate estimates of A, C, and E for each element arise from the multivariate optimization procedure. Supplemental Material, Table 3, shows that 20% of the variation in Zn concentration is estimated to be due to genetic factors (A) unique to Zn and another 6% to genetic factors shared with Cu; 14% is due to shared environment (C), of which 11% is ascribed to unique shared environment and 3% common to both Zn and Cu. For unshared environment effects (E), about half of the variation is unique and half shared with Cu. Overall, the unshared environmental variation is mainly unique for As, Pb, Hg, and Cd but shared by Cu, Zn, and Se. Shared (familial) environmental effects are negligible, and additive genetic effects are partly shared between As and Hg.

Estimation of the genetic correlations between pairs of elements (the correlation due to gene effects common to both) showed results similar to the phenotypic correlations in Table 1, except for the As/Hg pair, where the genetic correlation (*r*_G_) was 0.83 and the unique environmental correlation (*r*_E_) was 0.34. This shows that variation in some genes affects concentrations of both of these elements and to a greater extent than environmental variation affects both.

### Linkage analysis

**Figure 1:**
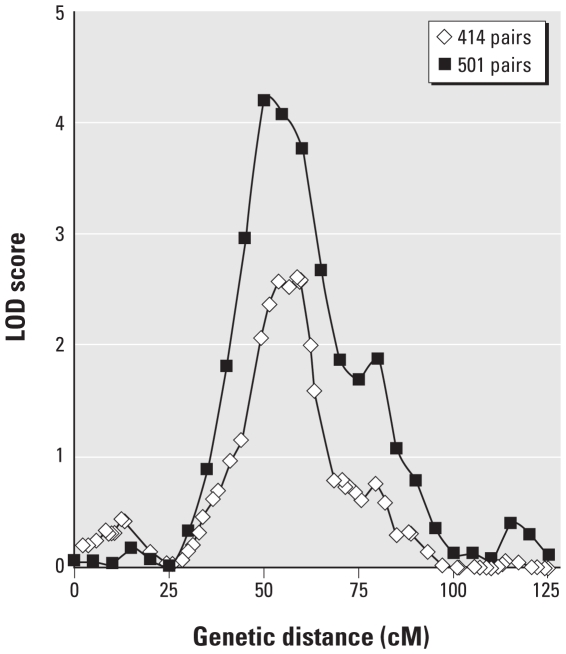
Details of linkage for Pb on chromosome 3. The increased number of DZ twin pairs [from 414 pairs reported previously (Whitfield et al. 2007) to 501] increased the peak LOD (logarithm of odds) score at 50 cM from 2.63 to 4.21.

**Figure 2:**
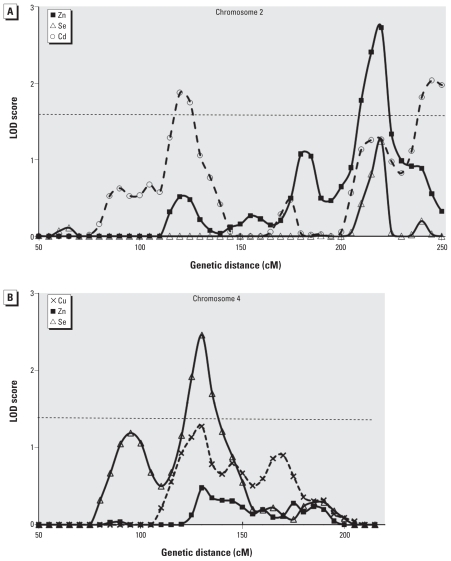
Linkage results for chromosomes 2 (*A*) and 4 (*B*), comparing results across elements. The horizontal line shows the suggestive LOD (logarithm of odds) threshold of 1.6.

Results for linkage analysis on 501 DZ twin pairs for three chromosomes are shown in Figure 1 (chromosome 3) and Figure 2 (chromosomes 2 and 4). Results for all chromosomes evaluated are summarized in Supplemental Material, Table 4 (doi:10.1289/ehp.0901541), and overall results are illustrated in Supplemental Material, Figure 2. As previously reported for 414 twin pairs (Whitfield et al. 2007), Pb gave the strongest linkage signal. The inclusion of extra twin pairs (for a total of 501 twin pairs) led to a revised LOD score of 4.21 on chromosome 3 (Figure 1), well within the significant range. The LOD score exceeded 4.21 in only 3 of 1,000 simulations, giving an empirical genome-wide *p*-value of 0.003. Suggestive linkage results (LOD > 1.6) were found for Zn on chromosome 2, Se on chromosomes 4 and 8, Hg on chromosome 5, and Cd on chromosomes 2, 18, 20, and X. There was some overlap of linkage signals for different elements (Figure 2; see also Supplemental Material, Table 4 and Figure 2), notably Cd and Zn around 120 cM and Zn, Cd, and Se at around 220 cM on chromosome 2 (Figure 2A); and Se and Cu around 130 cM on chromosome 4 (Figure 2B).

## Discussion

The main focus of this study was to assess whether, and to what extent, genetic variation between people affects individual differences in concentrations of toxic or trace elements. Using erythrocytes as a relevant and accessible model tissue, we identified genetic variation through use of twin data, comparing the similarity of members of MZ and DZ twin pairs. Where possible, we identified chromosomal locations of relevant genes through linkage analysis. Along with this genetic focus, we evaluated the effects of individual and socioeconomic factors. Incorporation of such effects in the data analysis also increases the power to detect and evaluate genetic effects. We found that genetic variation plays a significant role in determining concentrations of the measured elements, and some significant or suggestive genetic loci were identified by linkage analysis.

Many methods have been used to assess trace and toxic element status, although some are not applicable to humans. Easily available human materials that have been used previously are blood, urine, and hair, each of which has its uses and limitations. The primary limitation of the present study is that our results relate only to the concentration of measured elements in erythrocytes. For Pb, this is not a problem; measuring blood Pb is the standard procedure for evaluating current and recent exposure, and essentially all the Pb in blood is bound to erythrocytes. Plasma or serum Pb is < 1% of blood Pb (Manton et al. 2001; Schutz et al. 1996). Pb is not only bound to the erythrocytes, but also has direct effects on erythropoiesis, which can lead to anemia. For Cd, studies on Cd distribution in experimental animals show that injected Cd redistributes from the plasma to erythrocytes, and after 48 hr, erythrocyte Cd concentration is about 20 times the plasma concentration (Friberg et al. 1974). For Hg and As, the distribution between erythrocytes and plasma and the usefulness of urine measurements depend on speciation. Cu, Se, and Zn can be measured in plasma or erythrocytes (Vitoux et al. 1999), although erythrocyte Cu, Se, and Zn have the advantage of being less affected by the acute-phase response associated with inflammation or infections (Oakes et al. 2008).

### Subject characteristics and socioeconomic effects

As we expected, sex, age, and tobacco or alcohol use were significantly associated with several elements [Supplemental Material, Table 2 (doi:10.1289/ehp. 0901541)]. The most striking was a 3-fold increase in erythrocyte Cd in smokers compared with nonsmokers, which has been well documented (Grasmick et al. 1985; Moreau et al. 1983) and ascribed to selective Cd uptake by *Nicotiana* species (Lugon-Moulin et al. 2006). Cd concentration was also associated with sex (women had higher values) and educational level (higher levels of education were associated with lower values).

The quantity of alcohol consumed in the week before blood collection, as assessed from self-report of drinking, was positively associated with As, Hg, Pb, and Se concentrations [Supplemental Material, Figure 1 (doi:10.1289/ehp.0901541)]. There was also an apparent effect of alcohol on Cd, which may be due to the association between alcohol use and smoking; the association with alcohol was absent after adjusting for smoking status (Supplemental Material, Table 2).

We expected to find an association of alcohol with Pb based on results of other studies (Grandjean et al. 1981; Grasmick et al. 1985; Hense et al. 1992; Shaper et al. 1982) and our previous analysis of these data (Whitfield et al. 2007). However, there is little comparable information on the effects of alcohol on As or Hg. In a study on urinary As, Vahter and Lind (1986) found no effect of alcohol use, and in a study on urinary Hg in Hg-exposed dentists, Martin and Naleway (2004) observed lower excretion and, by inference, either lower absorption or greater retention among the drinkers. For Se, there are conflicting reports. Snook (1991) found that both plasma and whole-blood Se were significantly higher in light drinkers than in nondrinkers, whereas Ringstad et al. (1993) reported lower plasma Se in a group of men averaging 32 g alcohol/day than in abstainers. Lloyd et al. (1983) explicitly reported erythrocyte Se, which showed no detectable effect of drinking in apparently healthy volunteers. Results of the present study, covering a range of alcohol use from none to more than four drinks per day, suggest that alcohol is associated with slight but significant increases in Se and Hg and stronger increases in Pb and As; however, alcohol consumption appears to be associated with Cd only because of the association between alcohol use and smoking.

We also found some associations between measures of socioeconomic status and element concentrations. Increased time (years) of education was associated with decreasing As, Hg, and Pb concentrations. This association might be due to less occupational or residential exposure, but we observed no detectable effect of self-reported social class. It is likely that measured As and Hg concentrations are affected by seafood consumption (Balshaw et al. 2007; Borak and Hosgood 2007) and that much of the As or Hg is in the form of organic compounds, but we did not differentiate between organic and inorganic forms, nor did we have information on the diets of our subjects.

Another characteristic that might affect element concentrations is the use of mineral supplements. This could occur directly, for example, from Se or Zn in the supplements or indirectly from interactions between essential and toxic elements. Information from participants in another study conducted at about the same time, but restricted to people > 50 years of age, suggests that about 10% of these older participants were taking mineral supplements (Whitfield JB, Martin NG, unpublished data). However, we found no significant effect of mineral supplement use for any of the elements measured (data not shown). There were insufficient data on mineral supplement use to determine whether MZ twin pairs were more likely to be concordant than DZ twin pairs.

### Genetic influences

Twin-pair correlations for MZ and DZ twin pairs, after adjustment of the data for analytical variation, age, sex, smoking, alcohol use, and socioeconomic variables are shown in Table 2. For all the elements except Cd, the 95% CIs for MZ and DZ correlations do not overlap. This suggests the existence of genetic variation as a contributor to individual differences. We therefore proceeded to estimate the contributions of additive genetic effects (A), environmental effects shared within families (C), and nonshared environmental effects (E) on variation.

There was evidence of significant genetic effects on all of these elements except Cd (Table 2); even for Cd the 95% CI for additive genetic effects was 0–30%. These results are compatible with either genetic influences on behaviors that affect exposure [potentially including genetic effects on choice of diet (Hasselbalch et al. 2008), although there is no evidence for such effects on mineral content of the diet] or with genetic effects contributing to variation in uptake, retention, or distribution of these elements. Effects of shared environment (C) could not be demonstrated. Models in which shared environmental effects were excluded did not show a significant deterioration in fit to the data for any element, although effects in the range of 10–25% could not be excluded. Nonshared environmental effects (E) accounted for 60–80% of variation, including the effects of measurement error, day-to-day variation, and exposure to different environments and diets not shared by co-twins. However, and most important, the 95% CI for nonshared environmental effects excluded 100% for all elements measured; therefore, a purely environmental explanation for variation is not consistent with our data.

We next addressed the question of whether each element is subject to its own unique effects, or whether some genetic or environmental factors have common effects across several elements. This was examined in two stages: first by estimating the overall (phenotypic) correlation matrices for men and for women, and second, by estimating the genetic and environmental components of variation and covariation. We observed highly significant phenotypic correlations between concentrations of pairs or groups of elements, as shown in Table 1. In particular, results for Cu, Se, and Zn were strongly correlated, as were those for As and Hg. The correlations were almost identical in men and women. Such correlations could potentially be due to common effects on the analytical process, exposure to multiple elements present in contaminated environments, or to variation in metabolic processes affecting more than one element. Insight into these possible causes can be gained using the twin study method to distinguish genetic from environmental causes of variation.

Results of a multivariate analysis in which unique and common genetic and environmental effects were separately estimated are presented in Supplemental Material, Table 3 (doi:10.1289/ehp.0901541). Most genetic variation was unique to each element, shown by the largest effects being on the diagonal of the additive genetic matrix, except for the As–Hg element pair, which shows a substantial common (off-diagonal) effect. We found no evidence for shared environmental effects impacting more than one element. Nonshared environmental effects, which reflect both the aspects of the environment unique to each person (such as differing exposure) and analytical error, are element-specific, apart from some common effects between Cu, Zn, and Se. These common effects could be due to differences between people in their residential location or choice of diet, or to some people taking vitamin/mineral supplements containing all three elements, but we are unable to test for or distinguish between these possibilities.

The main point of interest in the multivariate analysis is the common genetic effect on As and Hg. These elements are not close chemically, but biologically each is found in organic compounds that may be absorbed as a result of eating fish or shellfish. However, we have no relevant data on diet for our subjects, still less on genetic influences on diet, and we cannot yet identify the reason for the genetic correlation between As and Hg concentrations. This may have to wait for identification of genetic loci or polymorphisms that affect both of them. More generally, there will be interactions between concentrations and toxic effects of the essential and toxic elements (Peraza et al. 1998) and between the elements reported here and iron status, but again, these will be easier to clarify once the main causes of variation in each single element are understood.

### Localization of genes by linkage analysis

The strongest linkage result for any of the seven elements was for Pb on chromosome 3, as previously reported (Whitfield et al. 2007). Availability of microsatellite marker data for extra pairs of DZ twins allowed a reanalysis, and the evidence for linkage increased from the previous LOD score of 2.63 to a highly significant 4.21 (Figure 1). The gene discussed in our previous article (Whitfield et al. 2007), *SLC4A7* (solute carrier family 4, sodium bicarbonate cotransporter, member 7), which codes for a transporter affecting Pb influx into erythrocytes, remains a strong candidate for harboring variation affecting erythrocyte Pb.

Two chromosomes showed linkage signals, although not at genome-wide significant levels, affecting more than one element [see Figure 2 and Supplemental Material, Table 4 (doi:10.1289/ehp.0901541)]. Chromosome 2 contained a region around 220 cM with a suggestive peak (LOD = 2.73) for Zn and minor peaks (each LOD ≈ 1.25) for Se and Cd. Chromosome 4 contained a region around 130 cM with a suggestive peak (LOD = 2.46) for Se and minor peaks for Cu (LOD = 1.28) and Cd (LOD = 0.56). Other regions containing peaks with LOD ≥ 1.6, the usual suggestive threshold for likely occurrence once in each genome-wide linkage analysis, were at 120 and 245 cM on chromosome 2 for Cd, 175 cM on chromosome 5 for Hg, 25 cM on chromosome 8 for Se, at the p-terminal end of chromosome 18 for Cd, around 15–30 cM on chromosome 20 for Cd, and on the X chromosome at 95 cM also for Cd.

Given these results, we can investigate whether genes likely to affect element transport or binding to proteins are located under any of these novel peaks. This excludes the chromosome 3 peak for Pb, which we discussed previously (Whitfield et al. 2007). Each peak is broad, as is common with genetic linkage analysis, and covers a large number of genes.

Starting with candidate genes expected to affect the processes of uptake, distribution, or binding of these elements, we checked their location against the regions showing suggestive linkage on chromosomes 2, 4, 5, 8, 18, 20, or X. The candidates include the hemochromatosis gene (chromosome 6), the metallothioneins (all chromosome 16), divalent metal transporters {*SLC11A1* [solute carrier family 11 (proton-coupled divalent metal ion transporters), member 1], chromosome 2; and *SLC11A2* [solute carrier family 11 (proton-coupled divalent metal ion transporters), member 2]*,* chromosome 12}, hepcidin (chromosome 19), and iron regulatory proteins [*ACO1* (soluble aconitase 1), chromosome 9; *IREB2* (iron-responsive element binding protein 2), chromosome 15]. The only overlap between a linkage peak and one of these candidate genes is for *SLC11A1* (at 218.9 Mb on chromosome 2), which is within the region of linkage for Zn on chromosome 2. The peak LOD score for Zn we found in the present study is 2.73 at 220 cM, or approximately 219 Mb. This gene codes for a divalent metal transporter that has been studied mainly in relation to iron, but the initial report on its characterization (Gunshin et al. 1997) clearly showed transport activity for Zn, Cd, Mn, Cu, and cobalt at rates similar to those for iron, and some transport of nickel and Pb. It is particularly interesting that the linkage region around 220 cM on chromosome 2 showed minor peaks for Cd and Se as well as the suggestive one for Zn, perhaps reflecting the broad specificity of this transporter.

Working in the other direction, starting with the locations of the suggestive linkage peaks and checking them against known genes in these regions, we could identify no other obvious candidates within the linkage regions.

### Strengths and limitations

Our data are derived from twin subjects. First, this is a major strength, because it allows the investigation of genetic and environmental sources of variation in individual elements and covariation between concentrations of two or more elements. Clearly, more can be inferred about the causes of covariation between elements when it is possible to ascribe it to either genetic or environmental effects on both. Second, use of related subjects (siblings; in this case, pairs of DZ twins) allows use of genetic linkage techniques to localize genes contributing to overall heritability. Twin studies have been questioned because of possibly greater similarity of environments in MZ than DZ pairs, but this is unlikely for our subjects, who were adults with a mean age of 46 years. Other limitations include restriction of the measurements to erythrocytes and lack of clinical data on our study participants. Given that the consequences of variation in toxic element concentrations in the general population are subtle, a focus on genetic effects on outcomes without prior consideration of intermediate phenotypes would require a prohibitively large study.

## Conclusions

The hypothesis underlying our studies of gene effects on toxic element concentrations is that although exposure is a precondition for harm, some people may be more vulnerable than others by reason of their greater tendency to absorb and retain one or several toxic elements. We have previously shown such variation for erythrocyte Pb concentration (Whitfield et al. 2007) and have now extended our analyses to conclude that the same applies for As, Hg, and probably for Cd. We have also identified genetic effects on Cu, Se, and Zn, which are not among the toxic elements but are essential and may modulate the toxic effects of the other elements. We now have one significant and several suggestive regions of linkage and have identified two strong candidate genes (*SLC4A7* and *SLC11A1*) for future association studies on single nucleotide polymorphisms.

Identification and confirmation of the genes and variants responsible depends on further research, but if such variants can be identified, then such data can be incorporated into models of the relationship between exposure and disease. Part of the difficulty in unraveling the health consequences of low-level exposure to toxic substances may be due to variation in vulnerability, and if a subgroup of the population can be defined as high risk, then exposure–disease associations may be found that are obscured in the general population. It may also be possible to protect people identified as being at high risk by reducing their occupational or environmental exposure to potentially hazardous situations. This concept of personalized medicine—basing treatment, advice, or lifestyle modification on genetic data—has been much discussed and may have major effects on individual and population health, but its application to occupational or population health would require both evidence of benefit and discussion of the social or ethical issues.

## Figures and Tables

**Table 1:** Correlations between element concentrations after adjustment for analytical covariates.

Element	Sex	Cu	Zn	Se	As	Pb	Hg	Cd
Cu	Male	—						
	Female							

Zn	Male	0.60	—					
	Female	0.53						

Se	Male	0.43	0.49	—				
	Female	0.42	0.47					

As	Male	0.15	0.15	0.28	—			
	Female	0.15	0.17	0.25				

Pb	Male	0.23	0.21	0.11	0.09	—		
	Female	0.18	0.22	0.13	0.09			

Hg	Male	0.08	0.05	0.25	0.48	0.07	—	
	Female	0.04	0.07	0.27	0.46	0.06		

Cd	Male	0.08	0.06	−0.03	0.04	0.20	−0.02	—
	Female	0.02	0.03	−0.05	0.00	0.21	−0.02	

With ~ 2,900 subjects, any *r* value consistently > 0.07 for both men and women is highly significant (*p* < 0.001).

**Table 2:** Twin pair correlations by zygosity [correlation (95% CI)] and estimated proportions of variance [percent (95% CI)] due to additive genetic (A), shared environmental (C), and nonshared environmental (E) factors.

	Cu	Zn	Se	As	Pb	Hg	Cd
MZ pair	0.29 (0.22 to 0.36)	0.41 (0.34 to 0.47)	0.36 (0.29 to 0.43)	0.22 (0.14 to 0.29)	0.41 (0.34 to 0.47)	0.32 (0.25 to 0.38)	0.35 (0.28 to 0.41)
DZ pair	0.10 (0.02 to 0.18)	0.25 (0.17 to 0.32)	0.20 (0.12 to 0.27)	0.04 (−0.04 to 0.13)	0.17 (0.09 to 0.25)	0.13 (0.04 to 0.21)	0.25 (0.17 to 0.32)
A	28 (14 to 34)	30 (11 to 46)	31 (10 to 40)	19 (5 to 26)	40 (26 to 45)	30 (15 to 36)	19 (0 to 31)
C	0 (0 to 11)	10 (0 to 26)	2 (0 to 18)	0 (0 to 11)	0 (0 to 11)	0 (0 to 12)	5 (0 to 22)
E	72 (66 to 79)	60 (54 to 67)	67 (60 to 74)	81 (74 to 88)	61 (55 to 67)	70 (64 to 77)	76 (69 to 84)
